# Reduced Platelet Aggregation and Plasma Cytokine Levels Mitigate Progressive Metabolic Dysfunction-Associated Steatotic Liver Disease (MASLD)

**DOI:** 10.2147/JIR.S533707

**Published:** 2025-08-20

**Authors:** Tobias Harm, Anne-Katrin Rohlfing, Jessica Kristin Henes, Nicole Manzat, Michal Droppa, Malte N Bongers, Mathias Heikenwaelder, Karin Anne Lydia Müller, Meinrad Paul Gawaz, Dominik Rath

**Affiliations:** 1 Department of Cardiology and Angiology, University Hospital Tübingen, Eberhard Karls University Tübingen, Tübingen, Germany; 2 Department of Diagnostic and Interventional Radiology, University Hospital Tübingen, Tübingen, Germany; 3 Division of Chronic Inflammation and Cancer, German Cancer Research Center Heidelberg (DKFZ), Heidelberg, Germany; 4 The M3 Research Institute, Eberhard Karls University Tübingen, Tübingen, Germany

**Keywords:** antiplatelet treatment, steatosis, coronary artery disease, chemokine signaling

## Abstract

**Purpose:**

Patients with metabolic syndrome and coronary artery disease (CAD) are at increased risk of metabolic dysfunction-associated steatotic liver disease (MASLD), which can progress to steatohepatitis, cirrhosis, and hepatocellular carcinoma. MASLD is the most common liver disease and a significant contributor to cardiovascular morbidity. Enhanced platelet aggregation is linked to steatohepatitis, and antiplatelet therapy has been suggested as a potential treatment.

**Patients and Methods:**

In a prospective study of 51 patients with type 2 diabetes mellitus and/or obesity (BMI≥30), we evaluated the impact of antiplatelet therapy on hepatic fat content, liver volume, and iron deposition using magnetic resonance imaging (MRI) at baseline and six months. Ex vivo platelet function testing and plasma levels of proinflammatory chemotactic cytokines were measured to characterize thromboinflammatory mechanisms underlying MASLD.

**Results:**

Increased platelet reactivity correlated with greater hepatic fat, iron deposition, and liver volume. Antiplatelet therapy was associated with reductions in hepatic volume and iron accumulation. Progression of steatosis was linked to dyslipidemia, platelet hyperreactivity, and elevated plasma levels of profibrotic, inflammatory, and apoptotic chemokines/cytokines. A distinct systemic cytokine profile corresponded with morphological features of progressive MASLD.

**Conclusion:**

Reduced platelet aggregation is associated with attenuation of MASLD features. Antiplatelet therapy correlates with decreased pro-inflammatory and pro-fibrotic chemokine signaling linked to the morphological characteristics of MASLD. Assessment of platelet reactivity and specific chemokines may enhance understanding of MASLD pathophysiology and support the development of novel therapeutic strategies.

## Graphical Abstract

**Figure:**
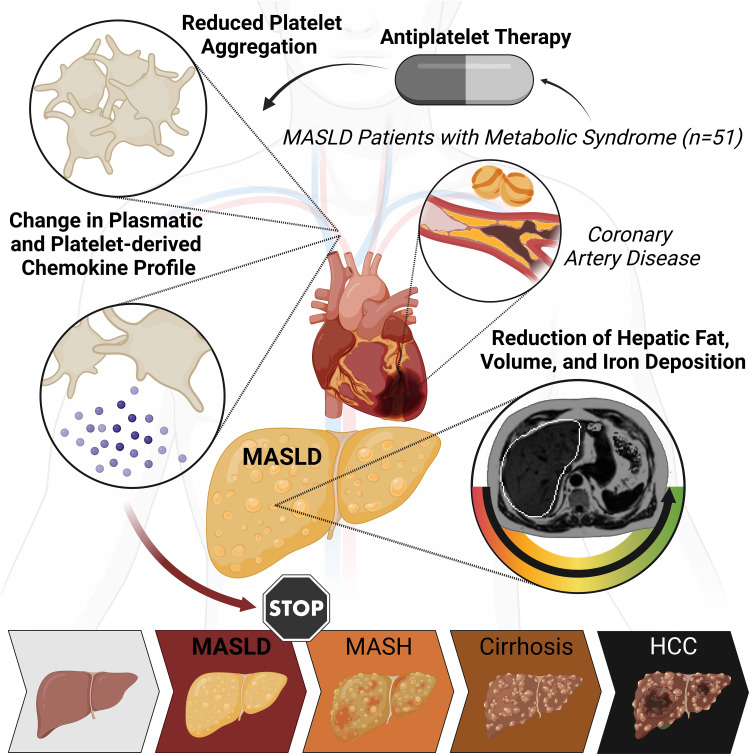

## Introduction

Platelets play a major role in hemostasis and platelet hyperreactivity drives atheroprogression leading to adverse cardiovascular events.^1–3^ Cardiovascular risk factors, including dyslipoproteinemia and diabetes, contribute to platelet activation.^4^,^5^ Sustained platelet hyperreactivity drives thrombo-inflammation and in turn an inflammatory microenvironment contributes to dysregulated metabolism.^6–8^ Metabolic syndrome, characterized by obesity, high blood pressure, insulin resistance, and dyslipidemia, is a significant risk factor for metabolic dysfunction-associated fatty liver disease (MAFLD).^9^,^10^ This spectrum extends from steatosis (metabolic dysfunction-associated steatotic liver disease, MASLD) to steatohepatitis (MASH), which can potentially progress to cirrhosis and, ultimately, hepatocellular carcinoma (HCC).^11^ Currently, MAFLD is the most prevalent chronic liver disease, and HCC is the fastest-growing cancer in high-income countries.^12^ Beyond reliable risk factors, MASLD has been recognized as a major contributor to cardiovascular disease burden.^13^ In the steatotic liver, a shift towards a pro-inflammatory, pro-coagulant, and pro-atherogenic metabolism inevitably raises the cardiovascular risk.^14^,^15^

Thus, cardiovascular events serve as a major contributor to all-cause mortality in patients with MASLD.^16^ However, there are no approved medications that effectively and safely reverse steatosis, inflammation, or fibrosis.^17^

Emerging evidence has highlighted the role of platelets as active contributors to MAFLD and inflammation. We and others have discovered that platelet function is strongly influenced by circulating chemokines that interact with platelet surface receptors.^18–20^ Furthermore, preclinical studies have identified platelets as initiators of steatohepatitis, and platelet-leukocyte interaction promotes further hepatocellular damage.^21^,^22^ Moreover, platelet count, activation, and aggregation are elevated in MASH but not in the early stages, such as MASLD.^23^ Antiplatelet therapy decreased intrahepatic platelet accumulation and cytokine levels, subsequently lowering the risk of hepatocarcinogenesis.^23^ Only recently, preliminary trials suggest that antiplatelet therapy may reduce hepatic fat content in patients with MASLD.^24^ However, the impact of antiplatelet therapy on platelet aggregation, systemic inflammation, and subsequent progression of early MASLD has not been studied comprehensively. Therefore, we aimed to investigate changes in hepatic fat, iron content, and liver volume following antiplatelet therapy in patients with MASLD. Subsequently, we elucidated alterations in chemotactic cytokine concentrations associated with the morphological traits of MASLD. Ultimately, we showed an association between cytokines and the progression of MASLD, which is possibly influenced by platelet activation.

## Materials and Methods

### Study Population

Fifty-one patients (n=51) with type 2 diabetes mellitus and/or obesity (body mass index; BMI≥30), both of which are significant risk factors and are strongly associated with the development of MAFLD, were consecutively enrolled into this prospective study (Table 1). All enrolled patients were admitted for the evaluation of cardiovascular disease (CVD), and invasive angiography was performed if coronary artery disease (CAD) was suspected, in accordance with current international guidelines (Supplemental Figure S1). Patients with symptomatic CAD (defined as at least one hemodynamically relevant, lumen-narrowing stenosis >50%) received single antiplatelet therapy (SAPT) with 100 mg of acetylsalicylic acid (ASA) once daily if CAD was present, but did not require coronary stent implantation. In case of percutaneous coronary intervention (PCI) and stent placement, dual antiplatelet therapy (DAPT) was administered, and included ASA 100 mg once daily and a P2Y_12_ inhibitor (clopidogrel 75 mg once daily, or ticagrelor 90 mg twice daily, or prasugrel 10 mg once daily) based on the acuity and severity of CAD. Patients without CAD evidence did not receive antiplatelet therapy. All patients adhered to the prescribed treatment regimen until follow-up. Post-hoc power analysis was performed based on the observed effect size (composite change in hepatic fat, liver volume, and hepatic iron content), comparing patients with and without antiplatelet therapy. Assuming a two-sided α=0.05, the estimated power for detecting the observed difference in the primary outcome was 81.9%. Patients were excluded from this study if they had received antiplatelet therapy prior to hospital admission. All patients completed a standardized questionnaire regarding medication history, smoking status, cardiovascular risk factors, and previous dietary factors. Furthermore, patients received lipid-lowering therapy according to the current CAD guidelines and disease severity.^25^,^26^ This prospective observational study was conducted in accordance with the STROBE (Strengthening the Reporting of Observational Studies in Epidemiology) guidelines. The trial was approved by the local ethics committee of Tübingen (587/2016BO2) and all patients provided written informed consent. The experiments were performed in accordance with the highest ethical standards and in compliance with the Declaration of Helsinki.

**Table 1:** Baseline Characteristics of Patient Population

	All	DAPT	SAPT	Naive	p-value
	(n=51)	(n=12; 23.5%)	(n=18; 35.3%)	(n=21; 41.2%)	
Female, n (%)	15 (29.4)	2 (16.7)	5 (27.8)	8 (38.1)	0.422
Age, years (median, IQR)	60 (51–67)	60.5 (50.8–67.5)	53 (48.5–61.3)	65 (55.5–67.5)	**0.047**
**Cardiovascular risk factors and scores**					
Arterial hypertension, n (%)	37 (74)	12 (24)	18 (36)	20 (40)	0.858
Hyperlipidemia, n (%)	23 (46)	9 (75)	9 (50)	5 (25)	**0.021**
Diabetes mellitus, n (%)	14 (28)	5 (41.7)	3 (16.7)	6 (30)	0.317
Current smoking, n (%)	13 (26)	4 (33.3)	7 (38.9)	2 (10)	0.103
Gensini score (median, IQR)	5.5 (1.4–36)	43.3 (15.5–64.6)	5 (2–10.5)	1 (0–12)	**0.003**
MAST score (median, IQR)	0 (0–1)	0 (0–1)	0 (0–1)	0 (0–1)	0.646
**Medication**					
Acetylsalicylic acid, n (%)	30 (58.8)	12 (100)	18 (100)	0 (0)	**<0.0001**
Clopidogrel, n (%)	2 (3.9)	2 (16.7)	0 (0)	0 (0)	**0.034**
Ticagrelor, n (%)	4 (7.8)	4 (33.3)	0 (0)	0 (0)	**<0.001**
Prasugrel, n (%)	6 (11.8)	6 (50)	0 (0)	0 (0)	**<0.0001**
Angiotensin-converting enzyme inhibitors, n (%)	26 (51)	7 (58.3)	12 (66.7)	7 (33.3)	0.098
Angiotensin II receptor antagonists, n (%)	16 (31.4)	4 (33.3)	5 (27.8)	7 (33.3)	0.920
Aldosterone antagonists, n (%)	11 (21.6)	4 (33.3)	4 (22.2)	3 (14.3)	0.439
Ca channel antagonists, n (%)	17 (33.3)	4 (33.3)	7 (38.9)	6 (28.6)	0.845
β-blockers, n (%)	29 (56.9)	11 (91.7)	8 (44.4)	10 (47.6)	**0.020**
Diuretics, n (%)	19 (37.3)	5 (41.7)	6 (33.3)	8 (38.1)	0.894
Statins, n (%)	33 (64.7)	12 (100)	16 (88.9)	5 (23.8)	**<0.0001**
**Laboratory parameters**					
GFR-MDRD (mL/min/1.73m^2^) (median, IQR)	85.7 (74.4–98.5)	87.6 (73.8–116.5)	88.6 (71.6–98.1)	83.6 (75.2–96.3)	0.602
LDL-cholesterol (mg/dL) (median, IQR)	114 (92–145.8)	116 (71–153)	121.5 (94–138.5)	111 (86–159)	0.936
HDL-cholesterol (mg/dL) (median, IQR)	42.5 (34–54.3)	34 (28.5–43)	43.5 (34–57)	47 (38–65)	0.048
Triglycerides (mg/dL) (median, IQR)	135 (121–172)	132 (126.5–190)	150 (127.5–268.5)	134 (98–165)	0.195
Total cholesterol (mg/mL) (median, IQR)	179.5 (148–210.5)	175 (123.5–206)	179 (165.8–201.5)	180 (132–217)	0.812
Platelets (10^9^/L) (median, IQR)	230 (196–268)	219 (174.8–272.3)	245 (219.8–321.8)	223 (183.5–256)	0.107
International normalized ratio (median, IQR)	1 (1–1.1)	1 (1–1)	1 (1–1.1)	1 (1–1)	0.216
ALT (U/L) (median, IQR)	30 (20–37)	31.5 (26–42.3)	30 (19.5–39)	25 (19–33)	0.332
AST (U/L) (median, IQR)	22 (16.5–26)	24 (15–31.8)	22 (17.28)	22 (15.5–24.8)	0.764
AP (U/L) (median, IQR)	75 (65–85.5)	73 (62–92)	73 (64.8–92.3)	80 (65.5–82)	0.973
Gamma-GT (U/L) (median, IQR)	38 (26–45)	30.8 (20.8–45.3)	42.5 (28–50.3)	35 (20.5–41.5)	0.335
Total bilirubin (mg/dl) (median, IQR)	0.6 (0.5–0.7)	0.6 (0.4–0.8)	42.5 (28–50.3)	0.6 (0.5–0.7)	0.704
Cholinesterase (U/mL) (median, IQR)	9.5 (8.4–11.1)	10.2 (9.3–11.2)	9.5 (8.5–11.5)	9 (7.8–10.6)	0.216
Albumin (g/dl) (median, IQR)	4.1 (3.9–4.3)	3.9 (3.9–4.1)	4.1 (3.8–4.4)	4.1 (4–4.3)	0.227
**Baseline MRI parameters**					
Liver fat (%) (median, IQR)	9.5 (6.7–12.7)	8.4 (5.7–13.6)	10.5 (7.5–12.4)	10.9 (7.4–16.2)	0.673
Liver volume (mL) (median, IQR)	1722 (1543–2351)	1598.5 (1456–2413.8)	1922 (1592.5–2279.8)	1722 (1394–2351)	0.573
Liver iron deposit (T2*) (median, IQR)	44.1 (36.3–59.5)	50.3 (42.9–88)	37.9 (32.3–44.7)	45.6 (38.7–56)	0.052
**Coronary artery disease**					
Acute coronary syndrome, n (%)	15 (29.4)	8 (66.7)	7 (38.9)	0 (0)	**<0.001**
Chronic coronary syndrome, n (%)	15 (29.4)	4 (33.3)	11 (61.1)	0 (0)	**<0.001**

**Note**: Significant (p<0.05) values are highlighted in bold.

### Diagnostic Workup of Patients with Metabolic Dysfunction-Associated Steatotic Liver Disease (MASLD)

Patients underwent liver magnetic resonance imaging (MRI) and blood sampling at baseline and after a median follow-up period of six months (Supplemental Figure S1). To identify patients with signs of steatotic liver disease (SLD), we analyzed morphological criteria, including hepatic fat, increased hepatorenal density, nodular liver morphology, and indicators of portal hypertension (such as splenomegaly, ascites, or intra-abdominal varices). After confirmation of SLD, a standardized diagnostic approach was implemented to rule out other causes of steatosis besides MASLD, such as autoimmune hepatitis, viral hepatitis, alcoholic fatty liver disease (AFLD), or liver congestion. Therefore, patients diagnosed with alcohol use disorders based on the Michigan Alcoholism Screening Test (MAST) as well as those with a reduced left ventricular ejection fraction (LVEF<45%) or severe valvular heart disease were excluded from the study.

### Magnetic Resonance Morphological Characterization of Metabolic Dysfunction-Associated Steatotic Liver Disease (MASLD)

Liver morphology, fat content, and iron storage were assessed using contrast-enhanced cardiac magnetic resonance imaging (MRI) with a 3T scanner (Siemens Medical Systems, Erlangen, Germany) as described previously.^23^ The standard internal protocol for liver MRI includes a non-fat-saturated coronal T2 HASTE sequence and an axial T2 BLADE TSE with fat saturation and respiratory triggering. Additionally, axial diffusion-weighted images with b-values of 0, 50, and 800 s/mm² were obtained, along with a corresponding apparent diffusion coefficient map. Following contrast medium administration, dynamic acquisition of the contrast distribution was performed using three sequential T1 VIBE Dixon sequences. Three minutes after the contrast injection, an additional axial and coronal T1 VIBE Dixon sequence was acquired. Finally, hepatic fat content and iron storage were quantified using a commercially available native T1-weighted multi-echo Dixon sequence (LiverLab, Siemens Medical Systems), which enabled the computation of proton density fat fraction maps and R2* (s^−1^) values for liver iron storage. Inline liver segmentation was then performed to determine liver volume. Liver MRI scans were analyzed using advanced software packages, and the results were independently reviewed in a blinded manner by two experienced investigators.

### Ex vivo Platelet Aggregation Analysis

To test whether platelet function is associated with MASLD progression, we performed ex vivo platelet function analysis. Therefore, blood sampling was performed according to a standardized protocol from peripheral venipuncture.^27^ Ex vivo whole blood platelet impedance aggregometry was analyzed using Multiplate Analyzer (F. Hoffmann-La Roche Ltd., Basel, Switzerland) as described previously.^28^ Whole blood samples from patients enrolled in this study were collected within 48 hours after hospital admission and after initiation of antiplatelet therapy, as well as at the six-month follow-up. Hirudinized whole blood (300 µL) was stimulated with 20 µL of adenosine diphosphate (6.5 µM ADP), arachidonic acid (484 µM AA), collagen (3.2 µg/mL COL), or thrombin receptor-activating peptide (32 µM TRAP). Platelet impedance was measured over 6 minutes, and the results were quantified as area under the curve (AUC=AUxmin) of aggregation units (AU), as previously described.^4^ In addition, to test for overall platelet hyperreactivity, average AUC of impedance aggregometry (ADP, AA, COL, TRAP) was reported.

### Assessment of Plasmatic Proinflammtory Chemotactic Cytokines

To investigate whether systemic inflammation is associated with MASLD progression and whether it is responsive to antiplatelet therapy, we conducted multilevel ex vivo functional assays. Therefore, blood samples were collected at baseline and after six months of follow-up. Plasma chemokine levels were measured using a proinflammatory chemokine panel (LEGENDplex™ kit, BioLegend, San Diego, USA), which employs specific bead-based monoclonal antibodies against individual chemokines. The chemokines analyzed were CCL2 (MCP-1), CCL3 (MIP-1α), CCL4 (MIP-1β), CCL5 (RANTES), CCL11 (eotaxin), CCL17 (TARC), CCL20 (MIP-3α), CXCL1 (GROα), CXCL5 (ENA-78), CXCL8 (IL-8), CXCL9 (MIG), CXCL10 (IP-10), and CXCL11 (ITAC). Therefore, plasma from CPDA-citrated whole blood samples was processed according to the manufacturer’s instructions, and the concentrations are reported in pg/mL.

### Flow Cytometric Detection of Platelet-Derived Proinflammatory Mediators

For analysis of the interplay between platelet-derived chemokines and cytokine-like mediators, we performed flow cytometric analysis of platelet (p) surface expression of P-selectin (CD62P), pCXCR4, pCXCR7 (ACKR3), pCXCL12 (SDF-1), pHMGB1, and pTGF-β1.^29^ Therefore, CPDA-citrated whole blood samples were diluted 1:50 with phosphate-buffered saline (PBS) and incubated with the respective conjugated antibodies (eg, CD62P-FITC mAb (Beckman Coulter Life Science, Krefeld, Germany), HMGB1-PE mAb, TGF-β1-PE mAb, CXCR4-PE mAb, CXCR7-PE mAb, SDF-1-CFS mAb (all from R&D Systems, Wiesbaden, Germany) for 30 min at room temperature (RT). Platelets were selected using CD42b-PE mAb (Beckman Coulter). After staining, samples were fixed with 0.5% paraformaldehyde and analyzed by flow cytometry using a FACSCalibur (Becton-Dickinson, Heidelberg, Germany).

### Statistical Analysis

Baseline characteristics and ex vivo data were analyzed using R (R Foundation for Statistical Computing, Vienna, Austria) and JMP Pro (SAS Institute, Cary, NC, USA). Data normality was assessed using distribution curve analysis and the Shapiro–Wilk test. Continuous variables are presented as median with interquartile range (IQR) or mean with 95% confidence interval (95% CI), as appropriate. Non-normally distributed data were compared using the Mann–Whitney *U*-test, while normally distributed data were analyzed using Student’s *t*-test. Categorical variables are presented as counts and proportions and were compared using the chi-square test. Correlations between normally distributed data were evaluated using Pearson’s correlation coefficient (r), whereas Spearman’s rank correlation coefficient (ρ) was used for non-normally distributed data. A False Discovery Rate (FDR)-controlling procedure (Benjamini-Hochberg) was applied to adjust the significance levels (p<0.05) where applicable. For the analysis of platelet aggregation, patients were divided into equally sized quartiles based on the corresponding area under the curve (AUC). Pattern Hunter analysis and Venn diagrams of the associations between cytokine levels and MRI data were performed using the “MetaboAnalyst” R package.

## Results

### Baseline Characteristics

In the present study, we examined the effects of antiplatelet therapy on liver morphology, as well as plasmatic and platelet-derived cytokine levels, in a prospective observational cohort (n=51) of patients with metabolic dysfunction-associated steatotic liver disease (MASLD) and suspected CAD.

**Figure 1:**
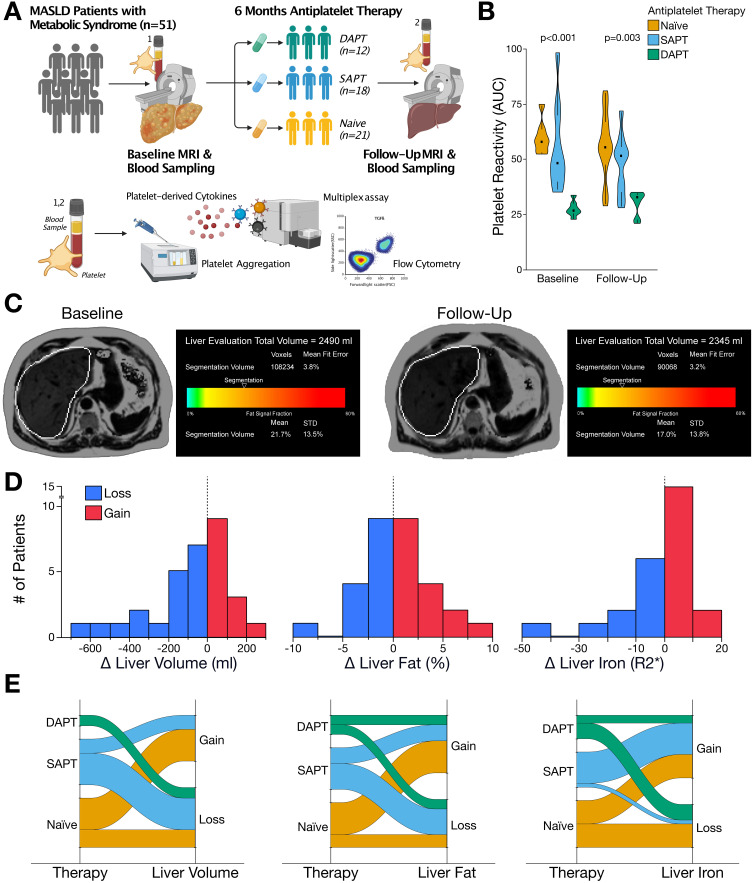
Morphological characterization of metabolic dysfunction-associated steatotic liver disease (MASLD) and the impact of antiplatelet therapy on disease progression. (**A**) Study flow-chart. Patients received six-months antiplatelet therapy according to CAD severity (eg, dual [DAPT], single [SAPT] or no antiplatelet therapy [naïve]). To determine the course MASLD, liver MRI was performed alongside assessment of platelet-derived mediators at baseline and after six-months follow-up. (**B**) Antiplatelet therapy, depending on its intensity, significantly (p<0.05) reduced platelet-hyperreactivity at baseline and after six-months of treatment, respectively. (**C**) Representative images of liver MRI assessing liver volume and hepatic fat content at baseline (left) and at six-months follow-up (right). (**D**) Bar charts showing the course of liver volume, hepatic fat content and iron storage. After six months of follow-up, most patients showed reduced liver volume, and iron accumulation, whereas hepatic fat content showed a heterogeneous trend. (**E**) Sankey-plots illustrating the course of liver volume, hepatic fat content and iron storage between patient receiving either no antiplatelet therapy, SAPT or DAPT. In the latter, liver volume, hepatic fat, and iron accumulation was predominantly reduced after six months-treatment.

The detailed workup of this study is shown in Figure 1A. The workflow started with coronary angiography if CAD was suspected, followed by antiplatelet therapy based on the disease severity (Supplemental Figure S1). MRI-based determinants of MASLD included quantification of hepatic fat content, iron storage, and liver volume at both baseline and after six months of follow-up. Similarly, laboratory markers of liver damage, as well as platelet-derived and plasma chemotactic cytokines, were assessed at baseline and at the six-month follow-up (Figure 1A). The patients’ baseline characteristics, including medical treatment and laboratory parameters at admission, as well as concomitant diseases, such as CAD severity, are summarized in Table 1. Among all patients, the baseline hepatic volume was 1722 mL (IQR 1543–2351 mL), hepatic fat content was 9.5% (IQR 6.7–12.7%), and relaxation rate correlating with iron storage was 44.1 s^−1^ (IQR 36.3–59.5 s^−1^). There were no significant differences in these parameters at baseline between patients who received mono- or dual-antiplatelet therapy and those who did not (Table 1).

### Morphofunctional Characterization of Metabolic Dysfunction-Associated Steatotic Liver Disease (MASLD) Among Patients with Antiplatelet Therapy

Recently, inhibition of platelet aggregation was shown to exhibit beneficial effects on the progression of MAFLD ex vivo.^23^ To investigate the effect of antiplatelet therapy on steatotic liver disease in a real-world setting, we enrolled patients with MASLD who were receiving either single antiplatelet therapy (SAPT, n=18), dual antiplatelet therapy (DAPT, n=12), or no antiplatelet therapy (naïve, n=21), depending on the presence and severity of CAD. We found that depending on the type of antiplatelet therapy, platelet aggregation was significantly reduced compared to that in naïve patients, both after initiation (p<0.0001) and six months of treatment (p=0.003) (Figure 1B).

To elucidate the course of MASLD, we performed MRI-based quantification of the hepatic volume, iron accumulation, and fat content. At baseline, we found that these morphological determinants were linked to advanced MASLD (Figure 1C). In addition, baseline liver volume and hepatic fat content at both baseline and after six months of follow-up were significantly correlated with hepatically predominant ALT, indicating liver damage and thus, prognostic relevance in patients with MASLD (Supplemental Figure S2).

However, after follow-up MRI, most patients showed a reduction of hepatic volume, iron accumulation, and fat content. However, a significant proportion of patients exhibited deterioration in all morphological parameters, indicating progression towards advanced MASLD (Figure 1D).

### Effect of Antiplatelet Treatment on Determinants of MASLD

When analyzing the dynamics of MRI parameters in the context of antiplatelet therapy, differences were observed between patients who received antiplatelet therapy and those who were naïve to antiplatelet therapy (Figure 1E). Patients who did not receive antiplatelet therapy predominantly exhibited increases in hepatic volume, fat, and iron content. Most patients receiving ASA monotherapy showed a reduction in liver volume and hepatic fat content but an increase in iron accumulation. Notably, all patients receiving DAPT showed a decrease in liver volume, and the majority exhibited reduced hepatic fat and iron content after six months of follow-up (Figure 1E).

**Figure 2:**
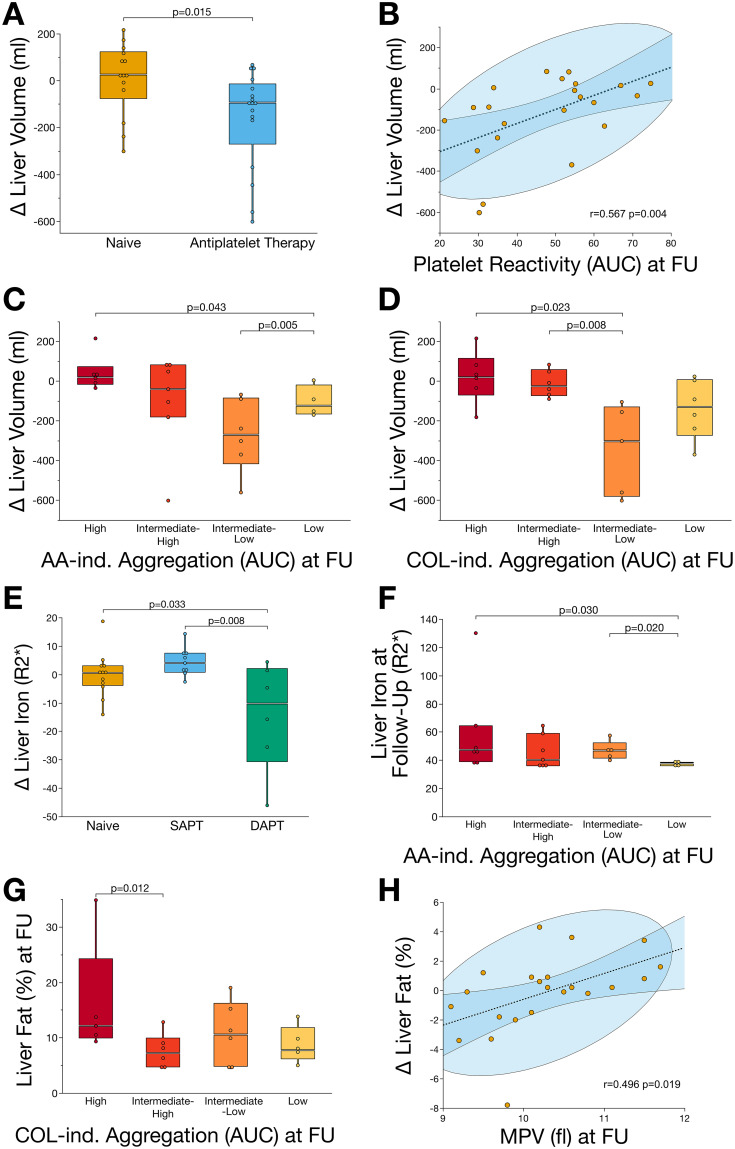
Decreased platelet reactivity resulting from antiplatelet therapy is linked to halting the progression of MASLD. (**A**) Patients receiving antiplatelet therapy showed a significant (p<0.05) reduction of liver volume after six-months treatment. (**B**) Reduced platelet aggregation significantly (p<0.05) correlated with reduced liver volume at follow-up. (**C**) Patients with low AA-induced platelet aggregation showed significantly (p<0.05) reduced liver volume compared to those with high reactivity. (**D**) Likewise, COL-induced platelet aggregation was significantly (p<0.05) associated with reduced liver volume at follow-up. (**E**) Antiplatelet therapy, depending on its intensity, was associated with a significant (p<0.05) reduction of hepatic iron storage. (**F**) After six-months of follow-up, patients with low AA-induced platelet aggregation showed lowest accumulation of hepatic iron when compared to patients with high platelet reactivity. (**G**) Hepatic fat content, the major determinant of steatotic liver disease, was significantly (p<0.05) enhanced in patients with high COL-induced platelet activity after six-months of follow-up. (**H**) Reduced MPV, indicating changes in platelet turnover and maturation following antiplatelet therapy, was significantly (p<0.05) associated with reduced hepatic fat content at follow-up.

**Abbreviations**: AA, arachidonic acid; AUC, area under the curve; COL, collagen; MPV, mean platelet volume.

To evaluate whether the influence of antiplatelet therapy on ex vivo platelet reactivity affects the course of MASLD, we analyzed alterations in MRI-based morphofunctional parameters. Here, we found that patients receiving antiplatelet therapy (SAPT and/or DAPT) exhibited a significant (p=0.015) reduction of liver volume after six months of treatment compared to naïve patients (Figure 2A). Furthermore, we found that reduced platelet reactivity was significantly correlated with reduced liver volume (r=0.567, p=0.004) (Figure 2B). To further investigate the impact of platelet reactivity on MASLD progression, patients were divided into quartiles according to the degree of platelet aggregation. At follow-up, patients with low arachidonic acid (AA)-induced platelet aggregation showed a significant (p=0.043) reduction in liver volume compared with patients with high platelet reactivity (Figure 2C). Likewise, patients with intermediate-low collagen (COL)-induced platelet aggregation at baseline showed significantly reduced liver volume compared to patients with intermediate (p=0.023) and high (p=0.008) platelet aggregation after six months of follow-up (Figure 2D).

Hepatic iron content was significantly associated with antiplatelet therapy regimen. In patients receiving DAPT, we found a significant decrease of iron storage when compared to patients receiving SAPT (p=0.008) or no antiplatelet therapy (p=0.033) (Figure 2E). Subsequently, patients with low AA-induced platelet aggregation showed reduced hepatic iron accumulation at follow-up when compared to those with intermediate-low or high platelet aggregation (Figure 2F). Similarly, hepatic fat content was associated with COL-induced platelet aggregation, as patients with high platelet reactivity showed enhanced steatosis at follow-up compared with those with intermediate to low platelet aggregation (Figure 2G). Interestingly, enhanced hepatic fat content was significantly correlated with mean platelet volume (MPV; r=0.496, p=0.019) indicating the potential role of platelet maturation in the progression of MASLD (Figure 2H).

### Antiplatelet Therapy Halts Inflammatory Pathways Driving MASLD Progression

**Figure 3:**
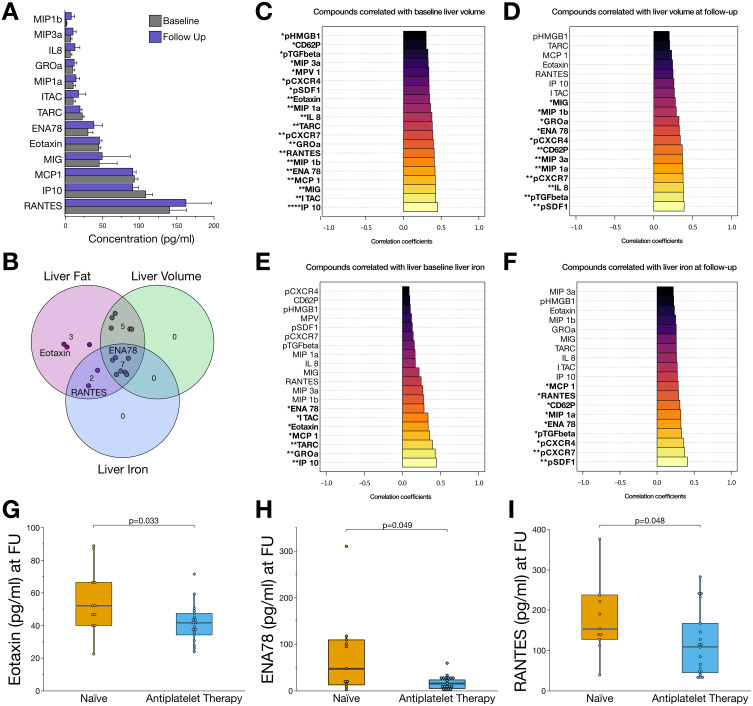
The plasma chemokine profile is influenced by the antiplatelet treatment and is associated with disease progression of patients with MASLD. (**A**) Bar charts showing concentrations of plasmatic chemokines at baseline and after six-months follow-up. (**B**) Venn diagram summarizing the most important chemokines that were significantly (p<0.05) associated with liver volume, hepatic fat content, and iron storage. Inflammatory mediators, which were susceptible to antiplatelet therapy in this study are labelled. (**C**) Bar charts of proinflammatory and profibrotic mediators significantly associated with liver volume at baseline. (**D**) Correlation analysis of mediators associated with liver volume at six-months follow-up. (**E**) Correlation analysis of circulatory and platelet-derived mediators associated with liver iron storage at baseline. (**F**) Correlation analysis of mediators and liver iron at six-months follow-up. (**G**) Concentrations of eotaxin (CCL11) were significantly reduced (p<0.05) in patients receiving antiplatelet therapy. (**H**) Among MASLD patients receiving antiplatelet therapy, concentrations of ENA-78 (CXCL5) were significantly reduced after six months of follow-up. (**I**) Concentrations of RANTES (CCL5) were reduced in patients receiving six months of antiplatelet therapy compared to those patients without antiplatelet therapy. *p<0.05, **p<0.01, ***p<0.001, ****p<0.0001.

To elucidate the impact of systemic inflammation on MASLD progression, we assessed the levels of platelet-derived chemotactic chemokines/cytokines and chemokine receptors on platelet surfaces. We found a wide dynamic range between distinct chemokine/cytokine concentrations, whereas the levels of individual mediators were relatively stable over time (Figure 3A). Furthermore, we performed comprehensive correlation analyses to elucidate the interplay between distinct platelet-derived systemic chemokines and the morphological determinants of MASLD (Figure 3B–F). The concentrations of these seven cytokines were consistently associated with liver volume, hepatic fat content, and iron storage (Figure 3B). In-depth analyses showed that liver volume and hepatic iron storage were associated with a distinct inflammatory fingerprint of chemokines/cytokines at both baseline and follow-up (Figure 3C–F). Furthermore, chemokine levels differed between patients with increased transaminase, AST, and ALT concentrations, suggesting a potential role in progressive liver damage in patients with MASLD (Supplemental Figure S3).

Plasma interferon gamma-induced protein 10 (IP10; C-X-C motif chemokine ligand 10 [CXCL10]), GROα (CXCL1), thymus- and activation-regulated chemokine (TARC; C-C motif chemokine ligand 17 [CCL17]), monocyte chemoattractant protein 1 (MCP1; CCL2), eotaxin (CCL11), interferon-inducible T-cell alpha chemoattractant (I-TAC; CXCL11), and epithelial-derived neutrophil-activating protein 78 (ENA78; CXCL5) levels were significantly (p<0.05) correlated with hepatic volume at baseline (Figure 3C) and follow-up (Figure 3D). Platelet surface stromal cell-derived factor 1 (SDF-1; pCXCL12), C-X-C motif chemokine receptors 4 (pCXCR4) and 7 (pCXCR7), transforming growth factor beta 1 (pTGF-ß1), ENA78, P-selectin (pCD62P), and plasmatic macrophage inflammatory protein 1b (MIP1b; CCL4) showed the strongest, significant (p<0.05) associations with hepatic iron storage at baseline and follow-up (Figure 3E and F).

**Figure 4:**
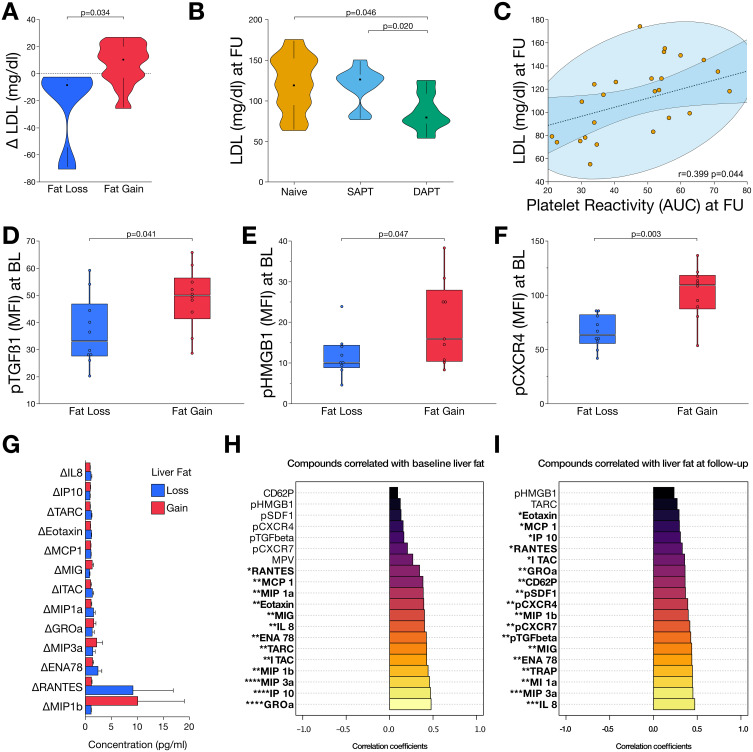
Diminished platelet reactivity and alterations in the fibro-inflammatory chemokine profile are associated with a reduction in hepatic steatosis. (**A**) Low-density lipoprotein (LDL) was significantly (p<0.05) increased in patients with progressive steatosis compared to those patients with reduced hepatic fat content. (**B**) After six months of antiplatelet therapy, LDL concentrations were significantly reduced (p<0.05), with the extent of reduction depending on the intensity of the treatment regimen. However, statin-treatment did not impact LDL concentrations significantly. (**C**) Reduced platelet reactivity significantly (p<0.05) correlated with low concentrations of LDL indicating an association between platelet reactivity and dyslipoproteinemia in this study. (**D**) In patients who exhibited progressive steatosis on MRI, baseline levels of profibrotic TGF-ß1 were significantly (p<0.05) increased. (**E**) Likewise, baseline levels of anti-apoptotic, angiogenic, and proinflammatory high-mobility group box protein 1 (HMGB1) were significantly (p<0.05) enhanced in patients with progressive MASLD. (**F**) Baseline levels of inflammatory pCXCR4 were significantly (p<0.05) elevated in patients with enhanced steatosis. (**G**) Bar charts depicting plasmatic chemokine concentrations at baseline and after a six-month follow-up, comparing patients with increased fat content and progressive steatosis to those with decreased hepatic fat content after six months of follow-up. (**H**) Correlation analysis showing that circulatory and platelet-derived mediators are associated with hepatic fat content at baseline. (**I**) Correlation analysis of fibro-inflammatory mediators and hepatic fat content at six-months follow-up. *p<0.05, **p<0.01, ***p<0.001, ****p<0.0001.

In the prospective analysis of chemokine profiles in patients with MASLD, we observed that the chemokine profiles of patients receiving antiplatelet therapy differed significantly from those of patients who were naïve to antiplatelet therapy (Figure 3G–I). After six months of antiplatelet therapy, we found significantly (p<0.05) reduced plasma concentrations of eotaxin, ENA78, and RANTES (Figure 3G–I). Notably, enhanced concentrations of the respective chemokines were linked to disease progression by showing a strong correlation with hepatic fat content, iron storage, liver volume, or transaminases (Figures 3C–F and 4H, I). Thus, changes in the plasma and platelet levels of distinct chemokines are associated with antiplatelet therapy and may modulate the course of MASLD.

### Reduced Platelet Reactivity Is Linked to Attenuation of Hepatic Steatosis

Hepatic steatosis is the major driver of MASLD and critically contributes to enhanced cardiovascular risk. In this study, we demonstrated a relationship between the progression of hepatic steatosis, dyslipoproteinemia, and platelet function (Figure 4). In patients who exhibited a reduction of hepatic fat content after six months of follow-up, we found reduced low-density lipoprotein concentrations that differed significantly (p=0.034) from enhanced LDL concentrations in patients who showed progressive steatosis (Figure 4A). Strikingly, we found that after six months of treatment with DAPT, LDL concentrations were significantly reduced compared to those in patients who received antiplatelet monotherapy (p=0.020) or no antiplatelet therapy (p=0.046) (Figure 4B).

The standard treatment for patients with metabolic syndrome often includes lipid-lowering therapy. To explore the potential confounding of statin-treatment on the impact of antiplatelet therapy on hepatic steatosis, we performed logistic regression analysis. Therefore, we included age, sex, baseline hepatic fat content, antiplatelet therapy, type of antiplatelet therapy (DAPT vs monotherapy/naïve), and new statin treatment in the model to assess the course of hepatic fat content. In multivariable regression analysis, we found that antiplatelet therapy, regardless of its type, was independently associated with reduced hepatic fat content (ß=−1.20, 95% CI: −2.34 to −0.07; p=0.038) (Supplemental Table S1 and Supplemental Figure S4).

However, we found that residual platelet aggregation was significantly correlated (r=0.399, p=0.044) with LDL concentrations after six months of antiplatelet treatment (Figure 4C). This implies that platelet reactivity determines the course of steatotic liver disease in this cohort. To further elaborate this hypothesis, we compared platelet-derived inflammatory mediators with respect to the course of hepatic fat content (Figure 4D–I). Interestingly, we found that the baseline levels of platelet-derived and profibrotic TGF-ß1 (p=0.041), anti-apoptotic, angiogenic, proinflammatory high-mobility group box protein 1 (HMGB1) (p=0.047), and inflammatory pCXCR4 (p=0.003) were significantly elevated in patients with enhanced steatosis (Figure 4D–F). Notably, plasma chemokine concentrations showed divergent dynamics after six months of follow-up, with RANTES exhibiting the strongest increase in patients with decreasing hepatic fat content and MIP1b in those with progressive steatosis (Figure 4G). Among the tested chemokines/cytokines associated with hepatic fat content at both baseline and follow-up, IL8, MIP1b, MIP3a, ENA78, MIG, GROα, I-TAC, RANTES, IP10, MCP1, and eotaxin showed a strong positive correlation (Figure 4H and I).

## Discussion

The major findings of the present study are as follows: 1) Platelet reactivity is associated with steatosis, liver volume, and hepatic iron storage in a cohort of patients with MASLD. 2) The administration of antiplatelet therapy was linked to the mitigation of MASLD, as assessed using MRI. 3) Plasma levels of chemokines and cytokines are correlated with the morphofunctional characteristics of MASLD. 4) Antiplatelet therapy is associated with reduced levels of profibrotic and inflammatory mediators. Our findings imply that platelet reactivity and levels of distinct plasma- and platelet-derived inflammatory mediators play a role in the pathophysiology and progression of MASLD. Antiplatelet therapy may be of potential value in limiting the progression of liver steatosis, especially in patients with CAD who require antiplatelet therapy for secondary prevention.

Over the past decades it has become evident that in addition to their central role in hemostasis, platelets are significant contributors to systemic and tissue inflammation.^3^,^6^ Platelets have been shown to play a potential important role in the pathophysiology of MAFLD in mice.^23^,^30^ Antiplatelet therapy has been suggested to mitigate development of fibrosis in MAFLD and thus potentially serving as an antifibrotic strategy to prevent progression to cirrhosis or HCC.^30–32^ Only recently, in a preliminary randomized clinical trial of patients with MASLD, six months of daily low-dose aspirin significantly reduced hepatic fat quantity.^24^ Previously, we and others demonstrated that platelet-derived mediators contribute to the progression of MAFLD to more advanced stages and promote cancerous effects in mice.^23^,^32^ However, the distinct interplay between antiplatelet therapy, platelet reactivity, systemic inflammatory responses and the progression of MASLD in humans remains unclear. Therefore, we hypothesized that platelet reactivity and changes of distinct plasma and platelet chemokines have an impact on liver steatosis. Our observation of an association between distinct chemokines/cytokines and hepatic fat content, iron accumulation, and liver volume suggests that systemic inflammation contributes to the pathophysiological cascade of MASLD. We demonstrated that platelet hyperreactivity and antiplatelet therapy were directly associated with reduced liver volume and decreased hepatic iron storage, predominantly in patients receiving DAPT. Hepatic iron overload triggers cell death through ferroptosis and lipid peroxidation, resulting from abnormal iron metabolism.^33^ Iron overload and ferroptosis play a role in triggering inflammation and the formation of steatohepatitis.^34^ Thus, a decrease of hepatic iron and mitigation of ferroptosis may become a therapeutic option to limit the onset or progression of MASLD. Our data support the concept that attenuation of platelet reactivity is associated with a decrease in systemic inflammation, which, in turn, limits liver remodeling with a reduction of liver volume (reduced fat accumulation and inflammatory swelling) and of iron overload, as verified by MRI.

Here, we found that distinct chemokines such as RANTES, eotaxin, and ENA78 were significantly reduced following antiplatelet therapy. Chemokines are directly associated with accumulation of hepatic fat, liver volume, and iron storage. ENA78 was recently shown to be upregulated during the genesis of HCC.^35^ In addition, eotaxin was lately revealed to be elevated in the development of MASLD and inhibition of eotaxin attenuated disease progression.^36^ Likewise, RANTES promotes liver-neutrophilic infiltration leading to MASH and HCC progression, whereas neutralization of RANTES attenuates hepatocarcinogenesis in a murine model.^37^ All three chemokines (RANTES, eotaxin, and ENA78) promote vascular inflammation and atherogenesis.^38–40^

Although we cannot provide direct evidence, it is tempting to speculate that downregulation of these systemic mediators following antiplatelet therapy might attenuate inflammation, and thus, the progression of MASLD. In addition, the anti-inflammatory effects of antiplatelet therapy may decrease the cardiovascular risk of patients with MASLD, metabolic syndrome, and cardiovascular disease. Of note, recent evidence has highlighted increased cardiovascular mortality in men with MASLD, mainly attributable to enhanced systemic inflammation, dyslipidemia, and insulin resistance.^41^,^42^ Furthermore, sex-specific differences in MASLD are influenced by visceral fat and hormonal changes, particularly in postmenopausal women.^43^ In our study, we did not observe significant differences in MASLD progression between genders. Additionally, antiplatelet therapy remained an independent predictor of hepatic steatosis after adjusting for sex, suggesting a potential benefit in both female and male patients.

Furthermore, dyslipidemia and elevated plasma lipoprotein levels play a pivotal role in MASLD pathogenesis by inducing hepatic lipotoxicity.^44^ Additionally, changes in platelet lipid metabolism contribute to platelet hyperreactivity, leading to an elevated cardiovascular risk.^4^,^27^,^45^ Therefore, accurate assessment of MASLD and the individual cardiovascular risk increasingly relies on a multi-step, non-invasive strategy. This includes initial blood-based scores such as the NAFLD fibrosis score (NFS) and fibrosis-4 index (FIB-4), followed by imaging modalities like transient elastography (FibroScan) and MR elastography to stage fibrosis, as well as MRI-based proton density fat fraction (PDFF) for quantifying hepatic steatosis.^46–48^ Additional biomarkers and composite scores, including the liver fibrosis score, and FibroScan-AST score can further refine risk stratification.^46^,^49^ In this context, our study demonstrated that antiplatelet therapy was associated with favorable changes in hepatic steatosis on MRI, alongside modulation of platelet-derived and systemic chemokines involved in thromboinflammation, suggesting a potential therapeutic benefit and novel biomarkers in MASLD.

Additionally, platelets have been shown to directly contribute to LDL metabolism and systemic inflammation.^50^ In this study, we outlined that, depending on the type of antiplatelet therapy, and subsequently, the degree of reduced platelet activation, plasma concentrations of LDL, as well as hepatic fat content were decreased following antiplatelet therapy. In multivariable analysis adjusted for statin treatment, antiplatelet therapy was significantly associated with reduced steatosis. In patients with decreased hepatic steatosis, we found lower baseline levels of profibrotic, proinflammatory, and pro-apoptotic platelet-derived TGF-1, HMGB1, and pCXCR4. Thus, the mitigation of MASLD following antiplatelet therapy may be associated with reduced LDL levels, as previously described. Therefore, in patients with CAD and an increased risk of MASLD, antiplatelet therapy might reduce cardiovascular risk, lead to resolution of steatosis, and improve the outcome of patients with MALSD.

## Limitations and Perspectives

We fully recognize the limitations of the present study with limited cohort size. While the sample size was relatively modest due to strict inclusion criteria, the study achieved an estimated power of 81.9% for the primary outcome, supporting the potential impact of antiplatelet therapy on thrombo-inflammation and MASLD progression. Nonetheless, we acknowledge that the selective nature of the cohort (type 2 diabetes and obesity) may reflect a collective at high cardiovascular risk and enhance internal validity of the findings but might also limit generalizability to broader MASLD populations. Although our follow-up period was limited to six months, this study highlights changes in MASLD after long-term antiplatelet therapy. In this study non-invasive MRI-based assessment of MASLD was aligned with current guideline recommendations to enhance clinical practicability. However, liver biopsy is considered the gold standard for MASLD diagnosis, and the lack of histological data in this study may limit pathophysiological interpretations. Furthermore, the main findings of this study, that antiplatelet therapy was associated with changes in thromboinflammatory signaling and disease progression of MASLD, were adjusted for important patient characteristics, including sex, comedication, and comorbidities. While antiplatelet therapy remained independently associated with the resolution of hepatic steatosis and changes in chemokine signaling, potential confounding effects of lipid-lowering therapy in this cohort cannot be fully excluded. In addition, the observational nature of this prospective study inherently limits causal inference. Thus, randomized controlled trials are warranted to further elucidate the direct impact of antiplatelet therapy on MASLD progression. Finally, future studies with longer follow-up periods are needed to assess long-term histological and clinical outcomes and to validate the observed improvements in hepatic steatosis and alterations in the chemokine profile following antiplatelet therapy. Nonetheless, we believe our findings offer a compelling hypothetical framework that combines functional and morphological parameters of steatosis, platelet reactivity, and systemic inflammation to elucidate associations with improved MASLD outcomes.

## Conclusion

Platelet hyperreactivity is associated with increased hepatic volume, iron deposition and fat content in patients with metabolic syndrome and MASLD. Dyslipidemia and a platelet-derived and plasmatic proinflammatory chemotactic cytokine profile promotes steatosis in patients with MASLD. Antiplatelet therapy is associated with less pro-inflammatory and pro-fibrotic chemokine signaling, thereby mitigating the disease progression of MASLD as observed on hepatic MRI. In patients with metabolic syndrome and MASLD receiving antiplatelet therapy for concomitant CAD, reduced platelet aggregation and altered chemokine signaling offer potential insights into the pathophysiology of steatotic liver disease. Furthermore, in CAD patients at high risk for MASLD, antiplatelet therapy may reduce the cardiovascular risk, promote steatosis resolution, and improve MASLD outcomes.
